# Radiologic response assessment in patients with desmoid-type fibromatosis treated with percutaneous cryoablation

**DOI:** 10.1186/s41747-026-00767-2

**Published:** 2026-07-01

**Authors:** Andrea Vanzulli, Lorenzo Saggiante, Lucilla Violetta Sciacqua, Tommaso Cascella, Carlo Spreafico, Giorgio Greco, Rodolfo Lanocita, Emanuele Rausa, Marco Vitellaro, Gabriele Tiné, Elena Palassini, Rosalba Miceli, Sandro Pasquali, Marco Fiore, Paolo Giovanni Casali, Silvia Stacchiotti, Alessandro Gronchi, Chiara Colombo, Carlo Morosi

**Affiliations:** 1 Department of Diagnostic and Interventional Radiology, Fondazione IRCCS Istituto Nazionale dei Tumori, Milan, Italy; 2 Department of Biomedical Sciences, Humanitas University, Pieve Emanuele, Milan, Italy; 3 Colorectal Surgery Unit, Fondazione IRCCS Istituto Nazionale dei Tumori, Milan, Italy; 4 Unit of Hereditary Digestive Tract Tumours, Fondazione IRCCS Istituto Nazionale dei Tumori, Milan, Italy; 5 Biostatistics for Clinical Research Unit, Fondazione IRCCS Istituto Nazionale dei Tumori, Milan, Italy; 6 Department of Cancer Medicine, Fondazione IRCCS Istituto Nazionale dei Tumori, Milan, Italy; 7 Department of Surgery, Fondazione IRCCS Istituto Nazionale dei Tumori, Milan, Italy; 8 Department of Experimental Oncology, Fondazione IRCCS Istituto Nazionale dei Tumori, Milan, Italy; 9 Department of Oncology and Hemato-Oncology, University of Milan, Milan, Italy

**Keywords:** Desmoid-type fibromatosis, Cryoablation, Magnetic resonance imaging, Radiology (interventional), Response evaluation criteria in solid tumors (RECIST)

## Abstract

**Objective:**

Percutaneous cryoablation (PC) has been incorporated among first-line treatment options for desmoid-type fibromatosis (DF). Loco-regional therapies, including PC, induce tissue changes that may precede measurable tumor shrinkage, thereby limiting the reliability of purely dimensional response criteria. To address this limitation, we compared response evaluation criteria in solid tumors (RECIST) 1.1 and magnetic resonance imaging (MRI)-adapted (M-)RECIST in patients with DF treated with PC.

**Materials and methods:**

We retrospectively identified all consecutive patients with progressing extra-abdominal DF treated with PC. Responses were assessed with RECIST 1.1 and M-RECIST, the latter relying on T2- and diffusion-weighted imaging, as well as unenhanced and contrast-enhanced T1-weighted imaging, to define residual viable tumor. Non-progression (NPR) and overall response rates (ORR) were defined as the percentage of patients without radiological progression and partial/complete responses, respectively.

**Results:**

Thirty-four patients (females/males, 26/8) and 37 procedures were identified. RECIST 1.1 and M-RECIST were applicable in 35 and 34 procedures, respectively. At a median follow-up of 15.7 months (interquartile range [IQR] 19.5), RECIST 1.1. Responses were: 10/35 (28.6%) partial response (PR), 22/35 (62.9%) stable disease (SD), and 3/35 (8.6%) progressive disease (PD), with ORR 28.6% and NPR 91.4%. At a median follow-up of 16.0 months (IQR 20.5), M-RECIST responses were: 15/34 (44.1%) complete response (CR), 12/34 (35.3%) PR, 4/34 (11.8%) SD, and 3/34 (8.8%) PD, with ORR 79.4% and NPR 91.2%. Overall concordance was negligible.

**Conclusion:**

M-RECIST yielded higher NPR/ORR than RECIST 1.1. These findings pave the way for studies addressing whether this shift in response categorization associates with improved outcomes prediction.

**Relevance statement:**

This work provides supporting evidence for the implementation of residual viable disease assessment in evaluating DF responses to PC.

**Key Points:**

- In patients with DF, responses to cryoablation can be characterized using purely dimensional and viability-based criteria.
- RECIST 1.1 and M-RECIST yielded complete agreement for PD.
- M-RECIST may refine response categorization below the progression threshold.

**Graphical Abstract:**

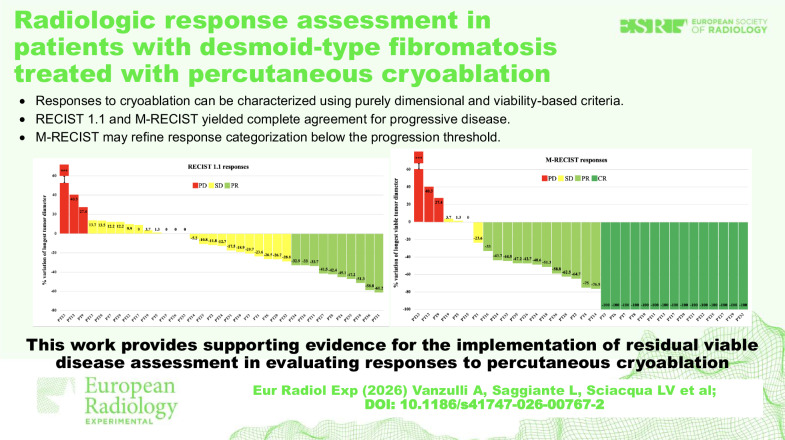

## Background

Desmoid-type fibromatosis (DF) is a rare (2–4 cases/million/year) fibroblastic proliferation arising from musculo-aponeurotic structures characterized by infiltrative growth, no metastatic potential and variable clinical behavior [1] Due to frequent spontaneous regression, active surveillance has been established as the upfront strategy, whereas treatment is reserved for DF that are threatening to life, function or quality of life or show persistent growth [1–10]

Percutaneous cryoablation (PC) is now considered a suitable first-line option for extra-abdominal DF requiring treatment, as part of a larger effort to shift towards nonsurgical management [11–19] Loco-regional therapies, including PC, elicit distinctive histopathological modifications that may not be accompanied by an immediate change in size, hampering the performance of purely dimensional radiologic response criteria.

Along these lines, response evaluation criteria in solid tumors (RECIST) have recently been proven suboptimal predictors of clinical outcomes in patients affected by high-risk soft tissue sarcomas of the trunk and extremities [20].

To overcome such limitations, we hereby propose a magnetic resonance imaging (MRI)-adaptation of the modified (m-)RECIST, which were originally developed for the assessment of treatment response in hepatocellular carcinoma [21–24]. In contrast to m-RECIST, which identify residual viable tumor exclusively through contrast-enhancement patterns, the hereby proposed MRI-adapted criteria (M-RECIST) leverage the multiparametric nature of MRI by incorporating in their evaluation T1-, T2- and diffusion-weighted imaging in addition to dynamic contrast-enhanced sequences.

The objective of this study was to compare overall response (ORR) and non-progression rates (NPR) as defined by RECIST 1.1 and M-RECIST through a retrospective evaluation of all consecutive patients with DF treated with PC at the Fondazione IRCCS Istituto Nazionale dei Tumori, Milan, Italy, between July 2021 and April 2025.

## Methods

### Study design

All consecutive patients affected by progressing (according to RECIST 1.1) extra-abdominal DF treated with PC at our institution between July 2021 and April 2025 were retrospectively reviewed.

### Informed consent and anonymization

Eligibility and fitness for the procedure were evaluated on a case-to-case basis by the institutional multidisciplinary tumor board. All patients provided written consent for the procedure. The study protocol was approved by the Comitato Etico Territoriale Lombardia 4, Milan, Italy (INT 156/25).

### Patient population and inclusion criteria

Patients affected by either primary or recurrent extra-abdominal DF arising in the head and neck, trunk, anterior abdominal and lumbo-sacral walls and extremities showing persistent RECIST 1.1 progression (confirmed at ≥ 2 consecutive follow-up appointments) during active surveillance or after other first-line treatments were treated with PC. Previous treatments included surgery, local-regional therapies (PC and irreversible chemo-electroporation), medical therapy (tamoxifen, methotrexate, vinorelbine, celecoxib, pregabalin, hydroxyurea) or a combination thereof (see also Supplementary Table S1).

Inclusion criteria were defined as follows: histological DF diagnosis confirmed by pathologists with dedicated experience on this pathological entity; availability of adequate baseline, intraprocedural, and post-treatment imaging; achievement of technical success, defined by the completion of the procedure.

### PC procedure

Procedures were performed under general anesthesia and computed tomography (CT) guidance by interventional radiologists with dedicated experience in the diagnosis and management of DF using an argon-based platform (CryoCare CS by Varian, Varian Medical Systems, Inc.). Number, size (1.7, 2.4 and 3.8 mm) and gauge of cryoprobes were decided on a case-to-case basis and tailored on lesion size, location and proximity to critical structures to balance radicality of treatment, safety and patient comfort. The correct positioning of the cryoprobes was always confirmed with CT before initiation of treatment.

Ablation volumes and safety margins were monitored in real time.

**Fig. 1:**
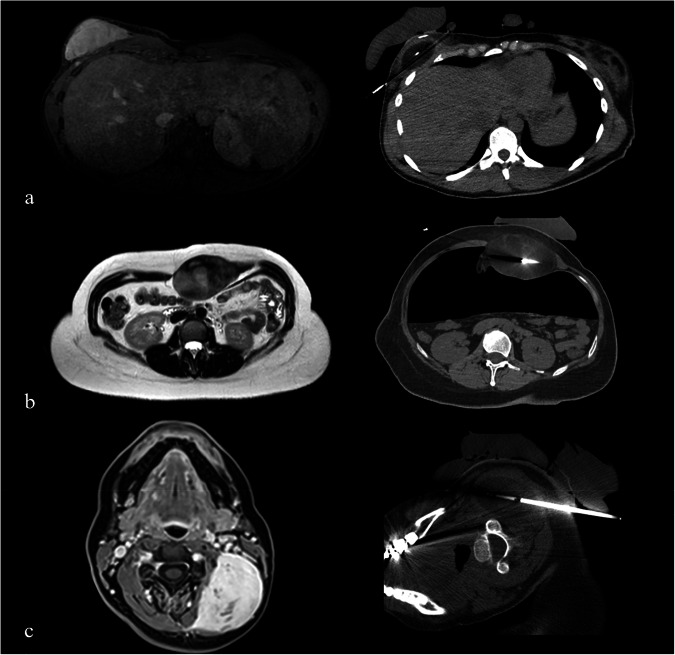
Safety measures to protect sensitive organs from heat-shock damage. **a** Proximity to sensitive structures. A DF arising in the subcutaneous adipose tissue of the anterior trunk wall, close to the skin. Sterile gloves filled with warm water and hydro-dissection of subcutaneous planes (please note the tip of the needle) were employed to protect the skin from heat-shock damage. **b** Proximity to sensitive structures: bowel. Pneumoperitoneum was induced with a Veress needle to distance abdominal viscera from the ice ball. **c** Proximity to sensitive structures: neuro-vascular bundles. Sterile gloves filled with warm water and safety margins were employed to protect the cervical neuro-vascular bundle from heat/cold-shock damage

Several strategies were employed to protect sensitive structures from heat/cold-shock damage (skin, bowel loops, abdominal viscera and neuro-vascular bundles), including the application of sterile gloves filled with warm water to the skin, hydro-dissection of subcutaneous planes with saline, intra-procedural neuromonitoring whenever operating close to neural plexuses or large nerves and the induction of iatrogenic pneumoperitoneum in patients with large DF of the abdominal wall using a Veress needle (Fig. 1).

### Imaging protocols

All patients included in this analysis had at least one preoperative MRI as part of their diagnostic workup, intra-procedural CT imaging to guide treatment and monitor the ablation volume in real time, and one or more re-evaluation MRI stored in our local Picture Archiving and Communication System‒PACS.

Tumor response assessment relied on a combined evaluation of unenhanced and contrast-enhanced acquisitions. Unenhanced sequences included: turbo spin-echo T1-weighted sequences; single-shot or turbo spin-echo T2-weighted sequences with and/or without fat signal suppression (short-tau inversion-recovery-STIR; spectral presaturation with inversion-recovery-SPIR; spectral-attenuated inversion-recovery-SPAIR; Dixon chemical shift-based fat-water separation; chemical shift selective-CHESS); diffusion-weighted sequences with high *b*-values, with corresponding apparent-diffusion coefficient-ADC maps. Contrast-enhanced acquisitions included T1-weighted turbo spin-echo or spoiled three-dimensional gradient-echo sequences (high-resolution isotropic volume examination-THRIVE, volumetric interpolated breath-hold examination-VIBE, or liver acquisition with volume acquisition-LAVA).

### Response assessment

Radiologic responses were assigned comparing the latest evaluable MRI examination against the baseline. Minimum and maximum follow-up times were 2 and 43.5 months, respectively. Baseline diameters were measured on the intra-procedural CT acquired before treatment initiation. Considering the inherent limitations of CT in depicting viable tumor tissue, we also reviewed the most recent preprocedural MRI to confirm viability.

**Fig. 2:**
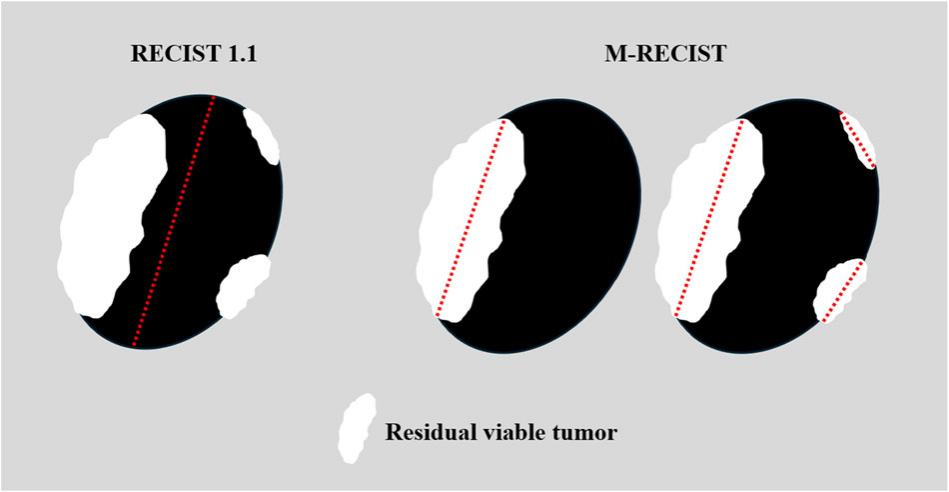
Conventional size-based (RECIST 1.1) and viability-adapted (M-RECIST) radiologic response criteria. Radiologic response assessment with RECIST 1.1 (left) and M-RECIST (right)

**Fig. 3:**
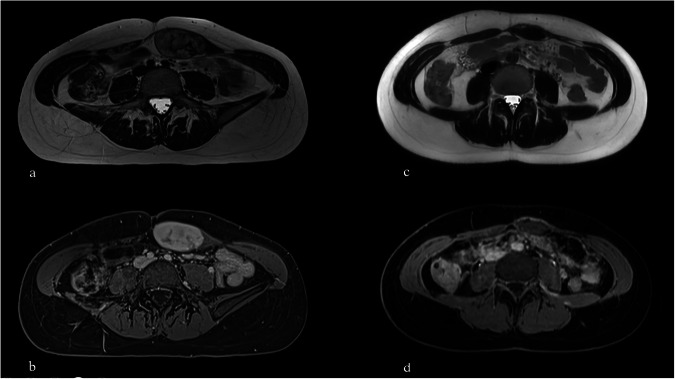
Complete response according to M-RECIST. **a** T2-weighted and (**b**) contrast-enhanced T1-weighted images of a DF arising in the anterior abdominal wall at baseline; **c** T2-weighted and (**d**) contrast-enhanced T1-weighted images after PC. The patient was classified as stable disease (SD) according to RECIST 1.1 due to subthreshold variation of the tumor’s longest diameter. The case was classified as complete response (CR) with M-RECIST due to diffuse fibrotic involution of the lesion, depicted by a marked decrease in T2 signal, and no evidence of nodular/mass-like enhancement after intravenous administration of gadolinium-based contrast agents; faint late enhancement of a perilesional fibrotic capsule, consistent with a CR, was also demonstrated

**Fig. 4:**
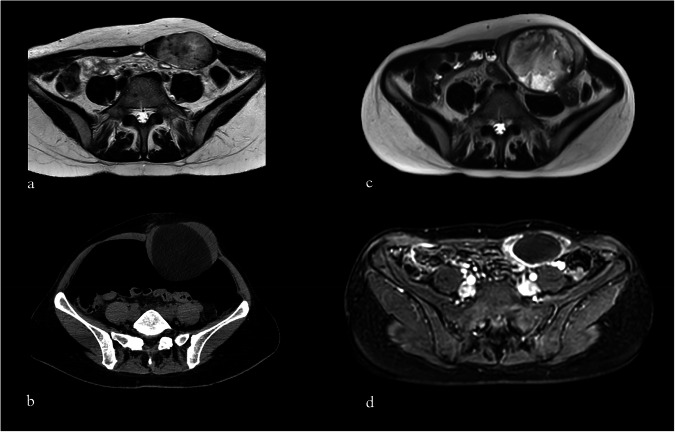
Partial response according to M-RECIST. **a** T2-weighted images at baseline; (**b**) intraprocedural CT imaging; (**c**) T2-weighted and (**d**) contrast-enhanced T1-weighted images after PC. Follow-up imaging (**c**, **d**) documented extensive colliquation of the lesion with persistence of peripheral soft tissue with intermediate T2 signal and late contrast enhancement, consistent with residual disease. The case was classified as SD according to RECIST 1.1 and partial response (PR) according to M-RECIST. CT imaging at the end of the procedure (**b**) already demonstrated undertreatment of the peripheral zone of the tumor

**Fig. 5:**
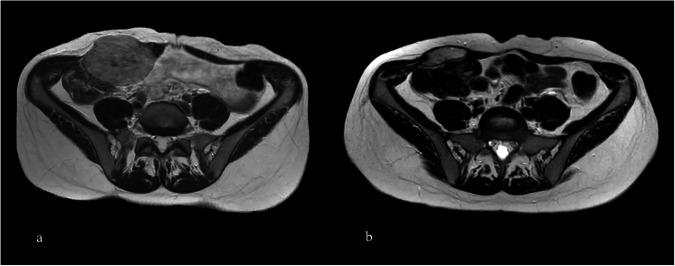
Response assessment with M-RECIST in patients with non-contrast MRI (complete response). For patients without adequate contrast-enhanced acquisitions at the latest available follow-up, tissue responses were assigned after a combined evaluation of T1, T2 and DWI changes after treatment, together with a retrospective evaluation of contrast-enhanced T1-weighted acquisitions from available priors. Follow-up imaging in this patient demonstrated extensive intralesional colliquation and a hypointense fibrotic capsule. The case was classified as CR according to M-RECIST and SD according to RECIST 1.1. **a** Baseline T2-weighted images; **b** T2-weighted images after treatment

**Fig. 6:**
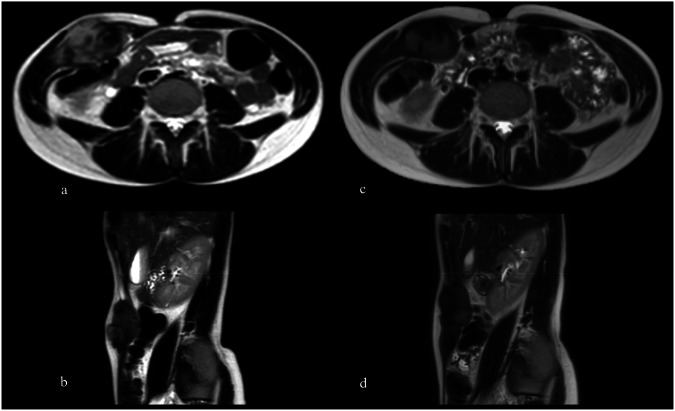
Response assessment with M-RECIST in patients with non-contrast MRI (progressive disease). For patients without adequate contrast-enhanced acquisitions at the latest available follow-up, tissue responses were assigned after a combined evaluation of T1, T2 and DWI changes after treatment, together with a retrospective evaluation of contrast-enhanced acquisitions at all available priors. T2-weighted images before (**a**, **b**) and after PC (**c**, **d**). Due to the persistence of pathological tissue, the case was classified as progressive disease (PD) with both RECIST 1.1 and M-RECIST

Residual viable tumor was defined via a combined analysis of signal changes in T1-, T2- and diffusion-weighted images, and, whenever available, T1 sequences after injection of contrast medium (Figs. 2–6). Images were centrally reviewed by two senior resident physicians (AV and LVS), one junior attending (LS) and a senior attending physician (CM) with dedicated experience in the diagnosis and management of DF (AV, LVS and LS approximately 3 years, CM > 30 years).

Radiologic responses were defined as follows:

- according to RECIST 1.1: percent variation of the longest diameter, defined as: (longest diameter after treatment *minus* longest diameter at baseline)/longest diameter at baseline × 100%;
- according to M-RECIST: percent variation of the longest viable tumor diameter, defined as: (longest viable diameter after treatment *minus* longest viable diameter at baseline)/longest *viable* diameter at baseline × 100%.

Complete response (CR) was defined as the disappearance of the lesion for RECIST 1.1 and by the absence of residual viable tumor for M-RECIST. Partial response (PR) was defined as a decrease of at least 30% in the longest (viable) tumor diameter, whereas progressive disease (PD) was defined as an increase of at least 20% in the longest (viable) tumor diameter or the appearance of new lesions. Stable disease (SD) was assigned in cases not qualifying for CR, PR or PD.

Responses were assigned by consensus, and discrepancies were resolved through joint review.

### Statistical analyses

NPR and ORR rates were defined as the percentage of patients without radiological progression and the percentage of patients achieving partial or CRs, respectively, according to each set of criteria.

The comparative evaluation of RECIST 1.1 and M-RECIST was performed in three complementary ways:

1. categorical agreement—agreement across the four ordered response categories (CR, PR, SD and PD) was quantified using Cohen κ with quadratic weights; two additional dichotomous categorizations (PD + SD *versus* PR + CR and PD *versus* SD + PR + CR) were assessed with unweighted Cohen κ;
2. continuous agreement—for each patient, the percentage change in the longest tumor diameter (∆%) was calculated with both sets of criteria, and the agreement between M-RECIST and RECIST 1.1 ∆% values was evaluated with Lin’s concordance correlation coefficient (CCC), along with 95% confidence interval;
3. Bland–Altman analysis, by plotting the difference against the average of the paired measurements; it provides an estimate of bias and the 95% limits of agreement (mean ± 1.96 standard deviations), which indicate the interval within which most differences between methods are expected to lie.

To support the interpretation of agreement in the presence of potentially unbalanced marginal distributions, the unweighted percent agreement was also reported.

As sensitivity analyses, chance-corrected agreement was also quantified using Gwet’s agreement coefficients, namely AC2 with quadratic weights for the four ordered response categories and AC1 for the dichotomous recordings [25].

All tests were two-sided and were considered statistically significant when yielding a *p*-value < 0.05. All analyses were conducted in R (version 4.4.1).

## Results

### Patients and procedures

Thirty-nine consecutive procedures were retrospectively identified. Technical success was achieved in 38/39 procedures; one additional patient was excluded from our analyses since histopathological review concluded for granulation tissue with no evidence of DF. The final cohort comprised 37 procedures performed on a total of 34 patients (26 females and 8 males, with a median age at treatment of 36 years, interquartile range (IQR) of 11), with one patient treated twice for two distinct lesions and two other patients each retreated for the same lesion at different timepoints. DF locations included the head and neck (2/37), trunk (11/37), anterior abdominal wall (18/37), lumbo-sacral wall (3/37) and extremities (3/37).

At the time of writing, follow-up imaging was not yet available for two patients (#30 and #37). No adequate post-treatment MRI was available for one patient (#31), who was thus only eligible for RECIST 1.1 evaluation on available CT imaging. Consequently, RECIST 1.1 and M-RECIST responses were assigned in 35 and 34 procedures, respectively. The median longest baseline diameter was 89 mm, with an IQR of 53 mm.

Nineteen patients were treated with PC due to persistent progression during active surveillance, whereas 18 procedures were performed following progression after other treatments, including medical therapy (11/18), PC (1/18), surgery (3/18), irreversible chemo-electroporation (1/18) or a sequence/combination thereof (2/18) (see also Supplementary Table S1).

For DF arising in the anterior abdominal wall (*n* = 18), iatrogenic pneumoperitoneum was induced in 7 procedures to protect viscera and bowel loops from heat/cold-shock damage. For 7/37 (19%) procedures, the latest evaluable follow-up study did not comprise adequate late T1-weighted acquisitions after intravenous administration of gadolinium-based contrast agents; M-RECIST responses were thus assigned based on a combined evaluation of T1-, T2-, and diffusion-weighted sequences alone.

### Radiologic response according to RECIST 1.1

**Fig. 7:**
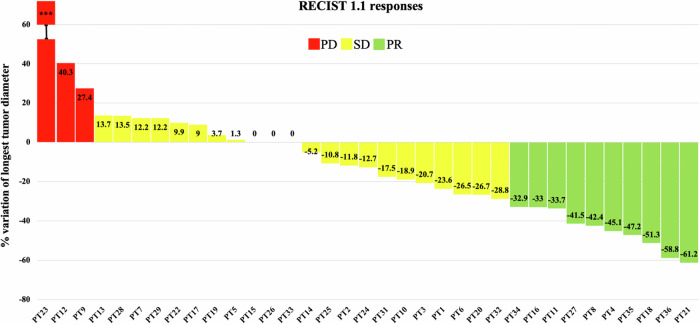
Radiologic responses according to RECIST 1.1 on the overall series of patients. Waterfall plot depicting RECIST 1.1 responses on the overall series of evaluable patients (*n* = 35). ^***^PD due to the appearance of new lesions adjacent to but distinct from the treated lesion

RECIST 1.1. responses were: 10/35 (28.6%) PR, 22/35 (62.9%) SD and 3/35 (8.6%) PD, accounting for an ORR and an NPR of 28.6% and 91.4%, respectively (median follow-up 15.7 months, with an IQR of 19.5). Median percent variation of the longest diameter was -15.1%, with an IQR of 36.1%. (Fig. 7)

**Fig. 8:**
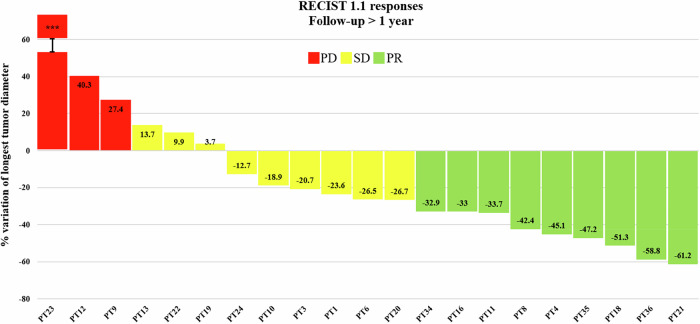
Radiologic responses according to RECIST 1.1 in patients with at least one year of follow-up. Waterfall plot depicting RECIST 1.1 responses on evaluable patients with at least one year of follow-up after the procedure (*n* = 20). ^***^PD due to the appearance of new lesions adjacent to but distinct from the treated lesion

Considering only patients with more than one year of follow-up (*n* = 21), RECIST 1.1 responses were: 9/21 (42.9%) PR, 9/21 (42.9%) SD and 3/21 (14.3%) PD, accounting for an ORR and an NPR of 40% and 90%, respectively (median follow-up 25 months, with an IQR of 16.8). Median percent variation of the longest diameter in this subgroup was -26.6%, with an IQR of 34.5% (Fig. 8).

### Radiologic response according to M-RECIST

**Fig. 9:**
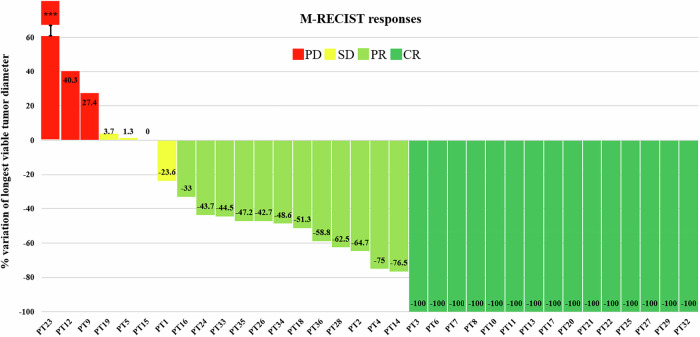
Radiologic responses according to M-RECIST on the overall series of patients. Waterfall plot depicting M-RECIST responses on the overall series of evaluable patients (*n* = 34). ^***^PD due to the appearance of new lesions adjacent to but distinct from the treated lesion

M-RECIST responses were: 15/34 (44.1%) CR, 12/34 (35.3%) PR, 4/34 (11.8%) SD and 3/34 (8.8%) PD, accounting for an ORR and an NPR of 79.4% and 91.2%, respectively (median follow-up of 16.0 months, with an IQR of 20.5). Median percent variation of the longest viable diameter was -75%, with an IQR of 55.5% (Fig. 9).

**Fig. 10:**
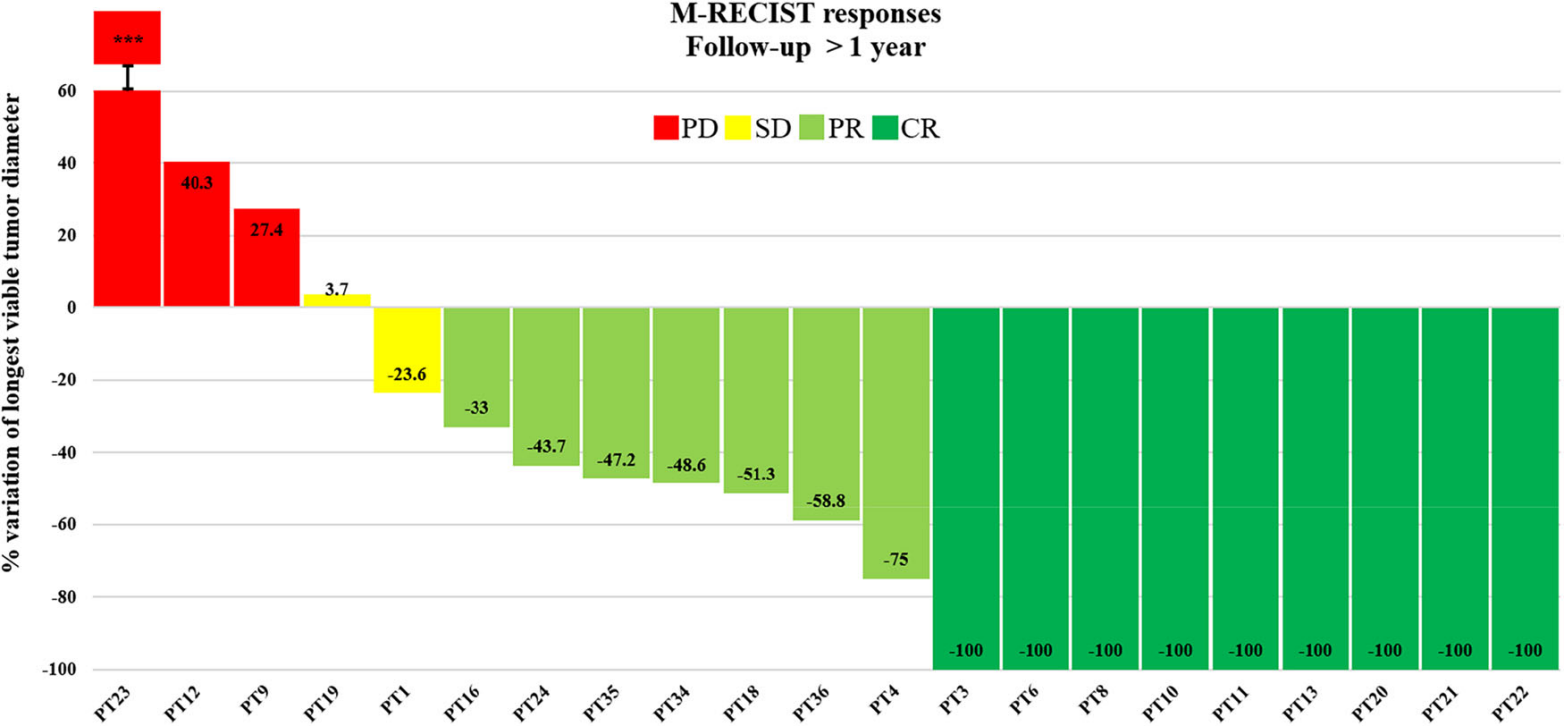
Radiologic responses according to M-RECIST in patients with at least one year of follow-up. Waterfall plot depicting M-RECIST responses on evaluable patients with at least one year of follow-up after the procedure (*n* = 20). ^***^PD due to the appearance of new lesions adjacent to but distinct from the treated lesion

Considering only patients with more than one year of follow-up (*n* = 21), M-RECIST responses were: 9/21 (42.9%) CR, 7/21 (33.3%) PR, 2/21 (9.5%) SD and 3/21 (14.3%) PD, accounting for an ORR and an NPR of 76.2% and 85.7%, respectively (median follow-up of 24.7 months, with an IQR of 17.3). Median percent variation of the longest viable diameter in this subgroup was -66.9%, with an IQR of 59% (Fig. 10). Patients displaying a CR at the first re-evaluation (performed approximately 30 to 40 days after treatment) showed sustained CR over time.

One patient (#23) was classified as PD according to both RECIST 1.1 and M-RECIST due to the appearance of a new lesion adjacent to the treatment area; notably, the treated DF showed a complete tissue response with extensive intralesional colliquation. For one patient (#10), the latest archived study documented the appearance of a vascularized nodule (longest diameter of approximately 18 mm) along the trace of the cryoprobe. The patient has still been assigned a CR according to M-RECIST due to complete colliquation of the treated lesion but will be granted closer follow-up to monitor and further characterize this finding, concerning for seeding. For one patient (#28), two distinct vascularized nodules were demonstrated at the periphery of the treated lesion at follow-up imaging; the longest viable residual diameter was computed as the sum of the longest individual diameters of the two nodules (see also Fig. 2).

No severe (Clavien-Dindo ≥ 3) post-procedural complications were recorded; the most frequently reported symptoms were edema of subcutaneous tissues in the ablated area and pain.

### Concordance analyses

The unweighted percent agreement across the four response categories (PD, SD, PR and CR) was 36.4%. (Supplementary Table S2). Bootstrap confidence intervals confirmed negligible overall agreement (quadratically weighted κ = 0.002; 95% bootstrap CI: 0-0.165). After collapsing the response data into two categories, agreement was low for PD + SD *versus* PR + CR (κ = 0.176; 95% bootstrap CI: 0.048–0.357; Supplementary Table S3) and perfect for PD *versus* SD + PR + CR (κ = 1; *p*-value < 0.001; Supplementary Table S4). Agreement for percentage change in longest tumor diameter (∆%) was poor: CCC was 0.16 (95% CI: 0.001–0.31), indicating substantial disagreement between the two criteria.

At sensitivity analyses using alternative chance-corrected agreement coefficients, results were consistent with Cohen κ values.

Across the four ordered categories, Gwet’s coefficients indicated poor agreement (AC1 = 0.174; 95% bootstrap CI: -0.126 to 0.394; and AC2 with quadratic weights = -0.214; 95% bootstrap CI: -0.661 to 0.302).

After dichotomization, Gwet’s AC1 similarly supported low agreement for PD + SD *versus* PR + CR (AC1 = -0.015) and perfect agreement for PD *versus* non-PD (AC1 = 1.000), reinforcing that discordance primarily concerned response grading within the non-progressive group rather than progression detection.

The Bland-Altman plot demonstrated a systematic negative bias of approximately -50% between the two criteria, indicating that M-RECIST consistently reported greater percent reductions than RECIST 1.1 (Supplementary Figs. 1 and 2).

## Discussion

In this single-center cohort of 34 consecutive patients with progressing extra-abdominal DF, remarkable NPR rates were achieved according to both RECIST 1.1 and M-RECIST.

Interestingly, the two sets of criteria were fully concordant in defining PD, yet RECIST 1.1 did not identify several partial and CRs—as defined by M-RECIST—and classified most patients as SD.

The complete concordance in identifying PD may support the notion that a dimensional increase beyond the 20% threshold represents a robust and unambiguous radiologic definition of progression, irrespective of nondimensional tissue changes. Conversely, when dimensional increase remains below such cutoff, agreement between criteria decreases, suggesting that M-RECIST may provide a more nuanced stratification of tumor responses compared with RECIST 1.1 in this subset of patients. Notably, patients with no residual viable disease at the first MRI after treatment (performed at approximately 30 to 40 days after cryoablation) showed no evidence of radiologic recurrence during follow-up, suggesting that M-RECIST CRs may be associated with enduring local control of disease.

This work investigates the role of PC in progressing extra-abdominal DF from a radiology-centered standpoint, with emphasis on post-treatment imaging assessment rather than clinical outcomes evaluation.

Consequently, the prognostic significance of this refined stratification remains to be further investigated and warrants validation in outcome-correlated studies. Our observations align with previously published data indicating potential limitations of RECIST 1.1, especially in the setting of loco-regional therapies [21–24, 26].

Similar attempts have been conducted in soft tissue sarcomas of the extremities and trunk wall, epithelioid hemangioendothelioma, gastrointestinal stromal tumors, hepatocellular carcinoma, and malignant pleural mesothelioma [20, 27–32].

Despite their known limitations, RECIST 1.1 remain the dominant framework for response assessment, largely due to their clarity and ease of use, which allow reproducible decision-making across readers and Institutions. However, the discretization of continuous biological processes into broad, fixed and somewhat arbitrary thresholds introduces an inherent loss of information and may obscure clinically meaningful trends.

In previous work, we advocated for a continuous-scale evaluation of dimensional change in patients with high-risk soft tissue sarcomas of the extremities and trunk wall treated with neoadjuvant chemotherapy [20].

We acknowledge such an approach may raise practical challenges, as continuous metrics lack intuitive clinical translation, complicate treatment stratification, and risk to overestimate measurement variability.

Consequently, efforts to replace or improve RECIST 1.1 should balance the gain in biological relevance with ease of use and interpretability.

This study has some limitations, notably its relatively small sample size, its retrospective design and the lack of a pre-planned correlation with clinical outcomes, the latter reflecting its primary focus on radiologic response assessment.

To the best of our knowledge, this represents the second largest single-center series of patients with progressing extra-abdominal DF treated with PC reported to date, and the third largest overall [11–18, 33].

To remove any selection bias, we included all patients with progressing extra-abdominal DF treated at the National Cancer Institute in Milan, Italy, in a defined timeframe.

Within contemporary management algorithms for DF, active surveillance represents the preferred initial strategy, whereas intervention is generally reserved for symptomatic and/or PD and should be discussed within a multidisciplinary setting. In this context, image-guided loco-regional therapies have emerged as organ- and function-preserving options for selected extra-abdominal DF.

Among minimally invasive techniques, PC offers several practical advantages, including real-time visualization of the ablation zone, the ability to shape conformal treatment volumes using multi-probe configurations, and the feasibility of adjunctive protective maneuvers when treating lesions adjacent to critical structures. In comparison, heat-based techniques such as radiofrequency or microwave ablation may be less amenable to direct intraprocedural depiction of the treated volume and may be more susceptible to heat-sink effects, while high-intensity focused ultrasound, although noninvasive, can be limited by acoustic windows, lesion accessibility, and treatment duration depending on lesion location.

The present study did not aim to compare the clinical effectiveness of different loco-regional therapies but rather aims to assess how different response assessment criteria may influence the interpretation of post-treatment imaging findings. DF are variably composed by a dense collagenous stroma—hypointense in both T1- and T2-weighted imaging—and an interspersed fibroblastic component, characterized by intermediate-to-hyperintense signal in T2-weighted images and representing active disease, able to drive disease progression and recurrence [34–36]. Preliminary evidence suggests that these two components may be accurately discerned with dynamic perfusion imaging, as the cellular component appears to display more aggressive kinetics of enhancement compared to the collagenous matrix [37].

In future work, we will integrate these and similar considerations to build (semi-)quantitative models to predict disease aggressiveness and clinical outcomes, including radiomics, which has already shown promising results in soft tissue tumors and other diseases [38–46].

## Supplementary information

**Additional File 1: Fig. S1** Concordance between standard and M- RECIST. In the Bland-Altman plot, the central solid line represents the mean difference, while the dashed lines denote the 95% limits of agreement, which span an extensive range from approximately -140 to +40. This wide interval highlights considerable disagreement at the individual level, demonstrating that the two techniques are not interchangeable. Furthermore, the dispersion of data points remains relatively uniform across the range of mean values, suggesting an absence of proportional bias. **Fig. S2** The Sankey diagram depicts the flow between standard and M-RECIST responses. Four cases of partial response were reclassified as CR, six cases of stable disease were reclassified as partial response and eleven cases of stable disease were reclassified as CR. **Table S1** Patients’ demographic and clinical characteristics. **Table S2** Confusion matrix (RECIST 1.1 *versus* M-RECIST **Table S3** Confusion matrix RECIST 1.1 *versus* M-RECIST for ORR rate. **Table S4** Confusion matrix RECIST 1.1 *versus* M-RECIST for NPR rate.

## Data Availability

All steps of the analysis are detailed in the body of the article. All additional information, including raw data, can be shared upon reasonable request to the corresponding author.

## Abbreviations

CR: Complete response
CT: Computed tomography
DF: Desmoid-type fibromatosis
IQR: Interquartile range
M-RECIST: MRI-adapted RECIST
m-RECIST: Modified RECIST
NPR: Non-progression rates
ORR: Overall response
PC: Percutaneous cryoablation
PD: Progressive disease
PR: Partial response
RECIST: Response evaluation criteria in solid tumors
SD: Stable disease
