# Supplementary material for: Radiologic response assessment in patients with desmoid-type fibromatosis treated with percutaneous cryoablation
Source: Eur Radiol Exp. 2026 Jul 1;10:102. doi: 10.1186/s41747-026-00767-2 (PMC13323459; doi:10.1186/s41747-026-00767-2)
Supplement: Supplementary file 1 — Additional File 1: Fig. S1 Concordance between standard and M- RECIST. In the Bland-Altman plot, the central solid line represents the mean difference, while the dashed lines denote the 95% limits of agreement, which span an extensive range from approximately -140 to +40. This wide interval highlights considerable disagreement at the individual level, demonstrating that the two techniques are not interchangeable. Furthermore, the dispersion of data points remains relatively uniform across the range of mean values, suggesting an absence of proportional bias. Fig. S2 The Sankey diagram depicts the flow between standard and M-RECIST responses. Four cases of partial response were reclassified as CR, six cases of stable disease were reclassified as partial response and eleven cases of stable disease were reclassified as CR. Table S1 Patients’ demographic and clinical characteristics. Table S2 Confusion matrix (RECIST 1.1 versus M-RECIST Table S3 Confusion matrix RECIST 1.1 versus M-RECIST for ORR rate. Table S4 Confusion matrix RECIST 1.1 versus M-RECIST for NPR rate. [file 41747_2026_767_MOESM1_ESM.pdf]

Radiologic response assessment in patients with desmoid-type fibromatosis treated with percutaneous cryoablation

ELECTRONIC SUPPLEMENTARY MATERIAL

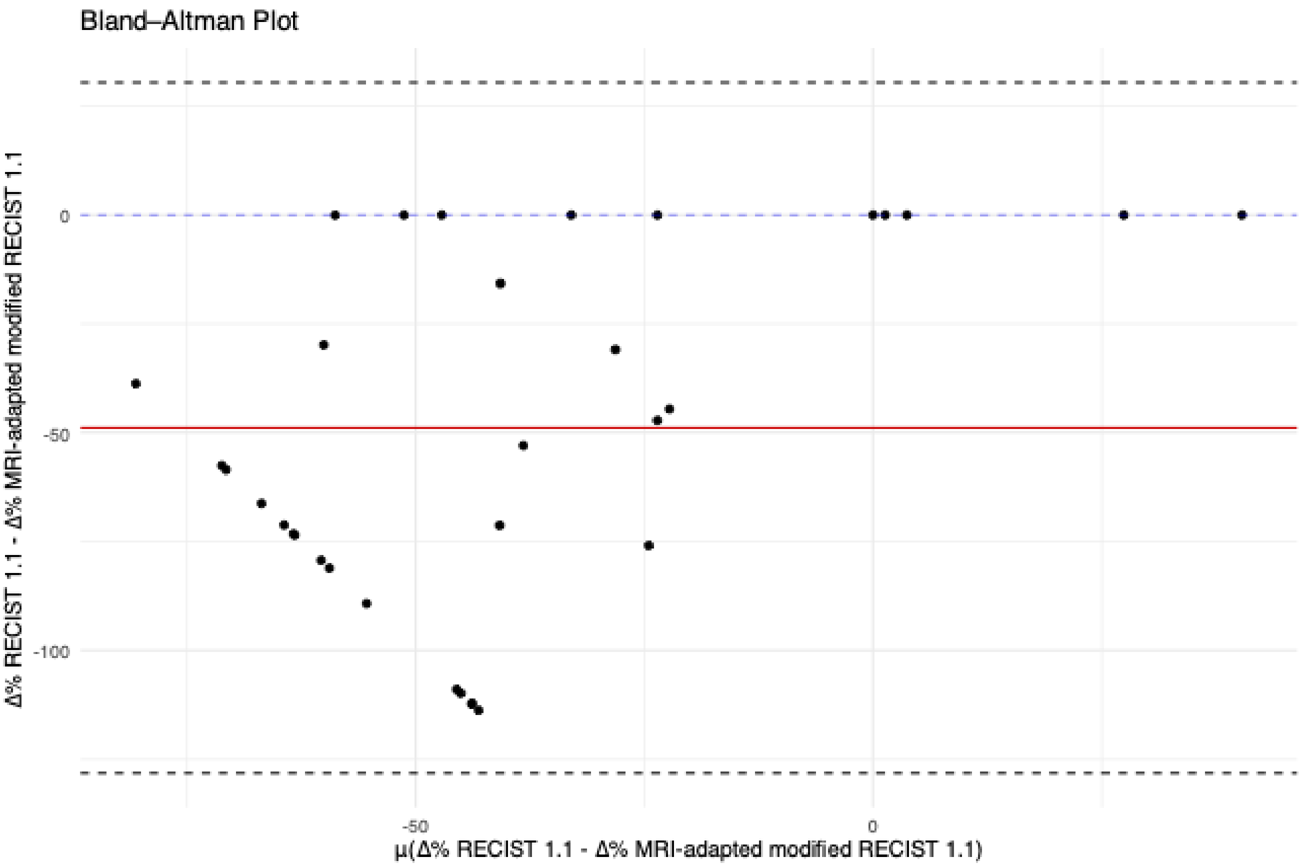

Fig. S1 Concordance between standard and M- RECIST. In the Bland-Altman plot, the central solid line represents the mean difference, while the dashed lines denote the 95% limits of agreement, which span an extensive range from approximately -140 to +40. This wide interval highlights considerable disagreement at the individual level, demonstrating that the two techniques are not interchangeable. Furthermore, the dispersion of data points remains relatively uniform across the range of mean values, suggesting an absence of proportional bias.

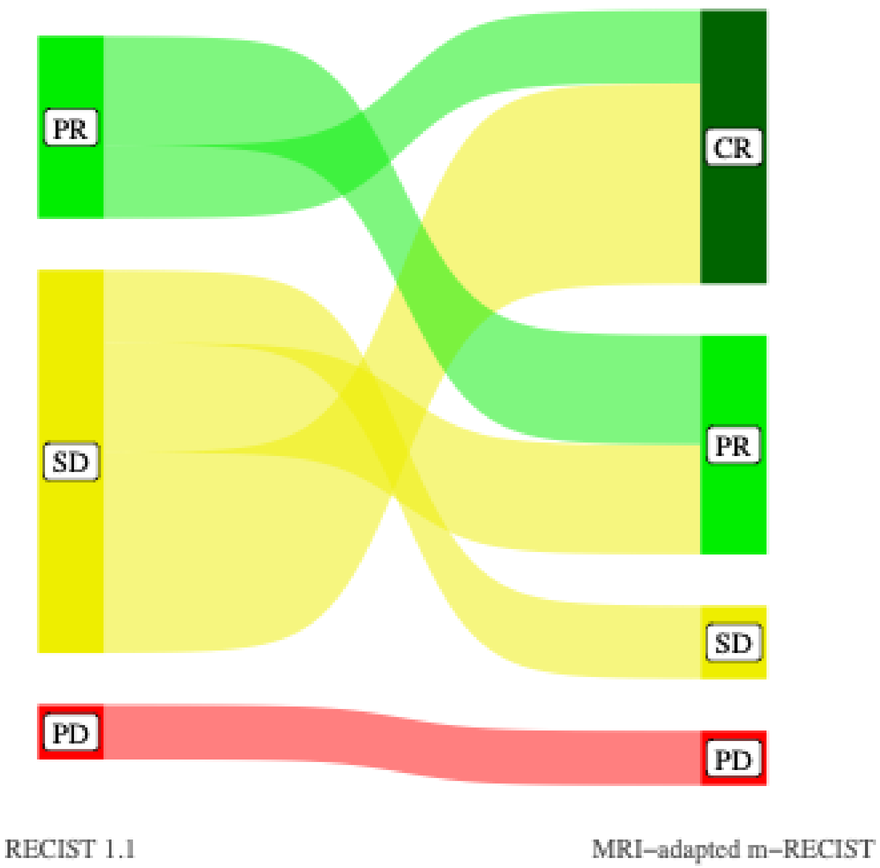

Fig. S2 The Sankey diagram depicts the flow between standard and M-RECIST responses. Four cases of partial response were reclassified as complete response, six cases of stable disease were reclassified as partial response and eleven cases of stable disease were reclassified as complete response.

Table S1 Patients' demographic and clinical characteristics

Treatment ID	Sex	Age at treatment (years)	Tumor location	Previous treatments	Longest diameter at baseline (mm)
PT1	F	25	Trunk	Active surveillance	89
PT2	F	27	Trunk	Percutaneous cryoablation (same lesion)	68
PT3	F	36	Anterior abdominal wall	Active surveillance	82
PT4	F	41	Anterior abdominal wall	Active surveillance + Tamoxifene	144
PT5	F	53	Head&Neck	Celecoxib + Methotrexate + Vinorelbine	77
PT6	F	36	Anterior abdominal wall	Methotrexate + Vinorelbine	68
PT7	F	38	Anterior abdominal wall	Methotrexate + Vinorelbine	49
PT8	F	39	Anterior abdominal wall	Active surveillance + Pregabalin	132
PT9	F	31	Lower extremity	Active surveillance + Tamoxifene	124
PT10	F	52	Anterior abdominal wall	Active surveillance	127
PT11	F	30	Anterior abdominal wall	Active surveillance	178
PT12	F	29	Anterior abdominal wall	Active surveillance	62
PT13	F	46	Lumbo-sacral wall	Surgery + Active surveillance	102
PT14	F	49	Anterior abdominal wall	Active surveillance + Methotrexate + Vinorelbine	153
PT15	M	13	Head&Neck	Active surveillance, Vinblastine + Methotrexate	133
PT16	F	29	Anterior abdominal wall	Active surveillance + Surgery	97
PT17	F	31	Anterior abdominal wall	Active surveillance + Surgery + Cryoablation	67
PT18	F	36	Anterior abdominal wall	Active surveillance	76
PT19	F	59	Trunk	Celecoxib	81
PT20	M	48	Trunk	Active surveillance	86
PT21	F	24	Trunk	Active surveillance	49
PT22	F	38	Anterior abdominal wall	Active surveillance	71
PT23	M	31	Lumbo-sacral wall	Surgery, Vinorelbine	96
PT24	F	40	Anterior abdominal wall	Active surveillance	126
PT25	F	41	Lumbo-sacral wall	Active surveillance	65
PT26	M	48	Trunk	Irreversible chemoelectroporation	125
PT27	F	22	Anterior abdominal wall	Active surveillance	82
PT28	F	39	Anterior abdominal wall	Active suirveillance	104
PT29	M	35	Trunk	Active suirveillance	98
PT30	F	30	Trunk	Celecoxib, tamoxifene, VBL + MTX, idrossiurea, pazopanib	76
PT31	M	33	Anterior abdominal wall	Active surveillance	97
PT32	F	37	Anterior abdominal wall	Active surveillance	59
PT33	F	35	Trunk	Active surveillance	110
PT34	M	57	Trunk	Active surveillance	146
PT 35	M	35	Upper extremity	Active survelliance	53
PT 36	F	55	Trunk	Tamoxifen, Celecobix	85
PT37	F	24	Upper extremity	Surgery	121

Table S2 Confusion matrix (RECIST 1.1 *versus* M-RECIST)

	**M-RECIST**			
**RECIST 1.1**	PD	SD	PR	CR
PD	2	0	0	0
SD	0	4	6	11
PR	0	0	6	4
CR	0	0	0	0

CR Complete response, *PD* Progressive disease, *PR* Partial response, *SD* Stable disease.

Table S3 Confusion matrix RECIST 1.1 *versus* M-RECIST for overall response rate

	**M-RECIST**	
**RECIST 1.1**	PD+SD	PR+CR
PD+SD	6	17
PR+CR	0	10

CR Complete response, *PD* Progressive disease, *PR* Partial response, *SD* Stable disease.

Table S4 Confusion matrix RECIST 1.1 *versus* M-RECIST for non-progression rate

	**M-RECIST**	
**RECIST 1.1**	PD	SD+PR+CR
PD	2	0
SD+PR+CR	0	31

CR Complete response, *PD* Progressive disease, *PR* Partial response, *SD* Stable disease.
